# Transmission of Foot-and-Mouth Disease SAT2 Viruses at the Wildlife–Livestock Interface of Two Major Transfrontier Conservation Areas in Southern Africa

**DOI:** 10.3389/fmicb.2016.00528

**Published:** 2016-04-22

**Authors:** Barbara P. Brito, Ferran Jori, Rahana Dwarka, Francois F. Maree, Livio Heath, Andres M. Perez

**Affiliations:** ^1^Foreign Animal Disease Research Unit, Plum Island Animal Disease Center, United States Department of Agriculture/Agricultural Research ServiceGreenport, NY, USA; ^2^Departamento de Medicina Preventiva Animal, Facultad de Ciencias Veterinarias y Pecuarias, Universidad de ChileSantiago, Chile; ^3^Unité Propre de Recherche Animal et Gestion Intégrée des Risques, French Agricultural Research Center for International Development (CIRAD)Montpellier, France; ^4^Department of Zoology and Entomology, University of PretoriaPretoria, South Africa; ^5^Department of Animal Science and Production, Botswana College of AgricultureGaborone, Botswana; ^6^Transboundary Animal Diseases Programme, Ondesterpoort Veterinary InstituteOnderstepoort, South Africa; ^7^Department of Veterinary Population Medicine, College of Veterinary Medicine, University of MinnesotaMinneapolis, MN, USA

**Keywords:** foot and mouth disease, SAT2, molecular epidemiology, phylogeography, Southern Africa

## Abstract

Over a decade ago, foot-and-mouth disease (FMD) re-emerged in Southern Africa specifically in beef exporting countries that had successfully maintained disease-free areas in the past. FMD virus (FMDV) serotype SAT2 has been responsible for a majority of these outbreaks. Epidemiological studies have revealed the importance of the African buffalo as the major wildlife FMD reservoir in the region. We used phylogeographic analysis to study dynamics of FMD transmission between buffalo and domestic cattle at the interface of the major wildlife protected areas in the region currently encompassing two largest Transfrontier conservation areas: Kavango–Zambezi (KAZA) and Great Limpopo (GL). Results of this study showed restricted local occurrence of each FMDV SAT2 topotypes I, II, and III, with occasional virus migration from KAZA to GL. Origins of outbreaks in livestock are frequently attributed to wild buffalo, but our results suggest that transmission from cattle to buffalo also occurs. We used coalescent Bayesian skyline analysis to study the genetic variation of the virus in cattle and buffalo, and discussed the association of these genetic changes in the virus and relevant epidemiological events that occurred in this area. Our results show that the genetic diversity of FMDV SAT2 has decreased in buffalo and cattle population during the last decade. This study contributes to understand the major dynamics of transmission and genetic variation of FMDV SAT2 in Southern Africa, which will could ultimately help in designing efficient strategies for the control of FMD at a local and regional level.

## Introduction

Foot-and-mouth disease (FMD) is one of the most important livestock diseases worldwide (OIE, World Organisation for Animal Health, 2009). FMD is caused by a single-stranded, positive sense RNA *Aphthovirus* from the *Picornaviridae* family. The genome has approximately 8,500 nucleotides (nt), of which ∼7,000 correspond to the open reading frame (ORF) that code for four structural proteins of the capsid and eight non-structural proteins. Among the capsid protein, VP1, which coded by 1D, is known to have important antigenic properties, it contains the RGD motif (Arg-Gly-Asp), which mediates the host cell receptor interaction in the GH loop. VP1 is the most variable protein-coding segment, and is the one traditionally used to study the distribution of the virus by looking at the sequence (Belsham, 2005; Carrillo et al., 2005).

Foot-and-mouth disease causes significant economic losses in endemic areas, and epidemics are devastating for livestock producers in disease-free countries due to their impact on beef exports restrictions (Perry and Rich, 2007; Knight-Jones and Rushton, 2013). Within Southern African countries, South Africa, Botswana, and Namibia have complied with the World Organization for Animal Health (OIE) standards and regulations, to certify FMD-free zones, where vaccination is not practiced (OIE, World Organisation for Animal Health, 2010). However, those countries struggle to control outbreaks originated from contacts with infected buffalo and illegal movements of livestock from neighboring countries. Banning of animal products trade due to FMD outbreaks results on far-reaching socio-economic consequences and, subsequently, impact on the development of the beef industry and society of affected countries (Scoones et al., 2010; Thomson et al., 2013b).

A unique feature of FMD epidemiology in Africa is the presence of the three South African Territories (SAT) serotypes FMD viruses (FMDV), namely, SAT1, SAT2, and SAT3, which are maintained within free ranging populations of African buffalo (*Syncerus caffer*). Interactions between infected buffalo and susceptible livestock result in FMDV transmission to livestock. However, the circumstances under which this transmission occurs remain poorly understood (Bastos et al., 2000; Sutmoller, 2002; Hargreaves et al., 2004; Jori et al., 2009). Specifically SAT2 serotype virus has been frequently detected from outbreaks in livestock (Bastos et al., 2003; Phologane et al., 2008).

Since the beginning of this century, transfrontier conservation areas (TFCA) have been established in Southern Africa to enhance biodiversity conservation and improve economic benefits of nature-based tourism and other related activities among rural communities living at the interface. The Kavango–Zambezi (KAZA) TFCA, and the Great Limpopo (GL) TFCA which encompass five (Botswana, Namibia, Zambia, Zimbabwe, and Angola) and three (South Africa, Mozambique, and Zimbabwe) countries, respectively, are the largest TFCAs in the region, including a total of 550000 km^2^ of protected areas. The ongoing creation of these TFCAs represents particular challenges at managing transboundary animal diseases (Thomson et al., 2013a). Those TFCA’s are home to large populations of African buffalo, while many rural communities live with their livestock at TFCA’s and surrounding areas (Brahmbhatt et al., 2011; Thomson et al., 2013a). The interaction between buffalo and livestock from surrounding communities has been described for some of the countries encompassed in these TFCA such as South Africa (Jori et al., 2009; Abu Samra et al., 2013), Zimbabwe (Caron et al., 2013; Miguel et al., 2013; Jori et al., 2016), Botswana (Eygelaar et al., 2015; Jori et al., 2015), and Zambia (Sinkala et al., 2014). Rural communities living on the outskirts of TFCA’s share common areas for pasture and water with other livestock owners and wild animals. The boundaries of protected areas can be delimited by a physical barriers such as a veterinary cordon fence or a river, allowing occasional contacts between cattle and buffalo (Jori et al., 2011). In other instances, physical barriers are entirely absent allowing regular contacts between both sympatric species, and the transmission of common pathogens, including FMD virus (Caron et al., 2013; Miguel et al., 2013). This situation renders the livestock/wildlife interface increasingly complex, a consequence that leads to the transmission of FMD to susceptible livestock (Brahmbhatt et al., 2011; Jori et al., 2016).

Previous studies of FMDV in the South African Development Community (SADC) attempted to assess the role of African buffalo in the epidemiology of FMDV and its transmission to domestic cattle at the wildlife–livestock interface by different approaches (Thomson et al., 1992, 2003; Jori et al., 2009; Brahmbhatt et al., 2011; Miguel et al., 2013; Jori and Etter, 2016). Although wildlife plays an important role in maintaining FMDV strains and occasional transmission to livestock and other wildlife species occur, some SAT viruses can also be maintained within livestock populations in countries where the disease has become endemic (Vosloo et al., 2002, 2009). However, dynamics of those transmissions and the importance of livestock or wildlife movements in the spread of FMD within sub-Saharan African countries have rarely been investigated (Tekleghiorghis et al., 2014).

To better understand the role of wildlife and livestock in maintaining and transmitting FMDV, we explored the transmission of FMD SAT2 virus between wildlife and livestock, at the interface of the main protected areas of Southern Africa using FMDV genetic data and phylogeographic analytical tools. Results here will contribute to elucidate the epidemiological dynamics of FMDV spread in Southern Africa and, ultimately, support disease prevention and control in the region.

## Materials and Methods

### Data Source

A total of 139 sequences obtained by the Onderstepoort Veterinary Institute (ARC-OVI) from Southern Africa (*n* = 57) and published in GenBank (*n* = 82) were used for the analysis. Sequenced viruses were obtained from vesicular lesions of clinically infected animals (domestic livestock and to a minor extent African buffalo) and buffalo probang samples, collected during surveillance programs between 1983 and 2012. Buffalo surveillance programs for FMD monitoring were mostly implemented in the late 1980’s and early 1990’s in Hwange National Park (HNP) in Zimbabwe and Kruger National Park (KNP) in South Africa, respectively. Cattle isolates were collected during outbreak investigations that occurred in different locations at the periphery of TFCAs. Sequence information and related epidemiological data (location, species, and date of collection) were organized in a database compiled by ARC-OVI (Supplementary Material).

Sequences used in the analysis (*n* = 139) were selected based on the availability of SAT2 virus sequence, information of the host (cattle or buffalo), and location. If the given location was within at most 50 km from the limit of a TFCA, it was assigned to the corresponding TFCA interface. Otherwise, the location was assigned to the country where the outbreak occurred.

### RT-PCR and Sequencing

Sequence analysis was performed at ARC-OVI, which is an OIE/FAO reference laboratory for FMD. The sequences corresponded to a partial VP1-coding segment of 384 nucleotides. The partial VP1-coding region was amplified using the WDA (Beck and Strohmaier, 1987) and VP1-AB (Bastos, 1998) primer set. This primer set was selected on the basis of its complementarity to the most conserved areas among different serotypes in the 2A/2B region, 33 nucleotides down-stream of the 3′ end of VP1-coding region. Direct DNA sequencing of amplicons yielded a consensus sequence representing the most probable nucleotide for each position. Sequences of the approximately 384 nucleotides of the VP1-coding region were compiled and edited using the BioEdit 5.0.9 software (Hall, 1999). The partial sequences correspond to nucleotide sites 265–648 of the complete VP1 coding segment, using SAT2 reference from published sequences (Bastos et al., 2003).

### Sequence Analysis

#### Phylogenetic Tree Model Selection

The 139 sequences were aligned using Multiple Sequence Alignment Comparison by Log-Expectation, implemented in the software MUSCLE (Edgar, 2004). To ensure the accuracy of the phylogenetic reconstruction, different parameters priors needed to be defined. First we determined the best codon partition scheme and respective substitution model for the sequences included in the study based on the Bayesian Information Criteria using PartitionFinder software package (Lanfear et al., 2012).

Alternative clock and tree priors were evaluated to determine the parameters that best described the tree topology of the FMDV analyzed. Clock models assessed were strict clock, lognormal uncorrelated, and exponential uncorrelated. Uncorrelated clock models allow specifying a parameter for the variation of the nucleotide substitution rate across lineages. Sequences were analyzed as serially sampled data, using the year in which the sample was collected. Each of the clock models was run using coalescent constant population size, coalescent exponential growth, and coalescent Bayesian skyline tree priors. The analysis was done using BEAST software v1.8.2 (Drummond et al., 2012). Bayesian analysis makes use of Markov Chain Monte Carlo (MCMC) methods to compute the posterior probability density of the tree parameters. We run the chains for of 5 × 10^8^ steps and sampled them every 10^4^. We checked the model for convergence so that all parameters reached an effective sample size >200. We annotated the maximum clade credibility (MCC) Tree burning the first 1000 trees generated or until convergence of the chain. The final tree model was selected using the posterior simulation-based analog of Akaike’s information criterion (AICM; Raftery et al., 2007). Model parameters were assessed for convergence and mixing using Tracer (Rambaut and Drummond, 2007). The final model was used to reconstruct the phylogeny of SAT2 viruses. All analysis were run using computational resources available in CIPRES Science Gateway Portal (Miller et al., 2010).

#### Discrete Traits for Host Species and Location

The model that best fitted the data was used to estimate the ancestral character reconstruction of the virus phylogeny based on location and species, utilizing these categories as partitions. An asymmetric Bayesian stochastic search variable selection (BSSVS) matrix was used to estimate non-zero rates of virus transmission between the categories of host and location (Lemey et al., 2009) implemented in BEAST v.1.8.2. A strict clock model for the traits was used. The significance of virus exchange between hosts and locations was assessed by computing Bayes Factors (BF) implemented in the software SPREAD (Bielejec et al., 2011), BF > 3.0 was considered significant. We used a Google Earth map image to display significant transmission between locations.

#### Estimation of the Viral Population Size, in the Context of FMD Relevant Events

Additionally, to compare estimated effective viral population size in time, we constructed a Bayesian Skyline plot for cattle sequences only, buffalo sequences only, and a separate one with all sequences. The effective viral population size approximates the viral diversity in time. We further compared the changes of the viral diversity with events relevant to FMD occurrence in southern areas in Africa:

– *From 1951 to 1965*: Most of the countries in Southern Africa- except South Africa- remained under colonial rule and begun implementing FMD control. Veterinary fences initiated their construction during this period (Scoones et al., 2010). In the meantime, FMD was controlled by aphthisation until 1963 (Derah and Mokopasetso, 2005).
– *Period 1965 to 1999:* Mid 1960’s was the beginning of independence for most of African countries (Botswana, Namibia, and Zimbabwe). In this period, FMD vaccines were introduced, combined with the erection of veterinary cordon fences by the 1970’s (Baipoledi et al., 2004). These methods showed their success since de mid 1970’s up to the beginning of the XXIth century. Indeed, during the last two decades of the XXth century, very few outbreaks were reported (Bruckner et al., 2002; Baipoledi et al., 2004; Thomson et al., 2013a; Jori et al., 2016), facilitating the creation of export or free zones with and without vaccination in South Africa (Bruckner et al., 2002), Zimbabwe (Derah and Mokopasetso, 2005), Botswana (Falconer, 1972; Baipoledi et al., 2004), and Namibia (Bishi and Kamwi, 2008).
– *From 2000 to current days:* Since the beginning the XXI century there was a dramatic change in the epidemiological situation on Southern Africa. First, the land reform and political turmoil in Zimbabwe, disrupted ongoing FMD control programs in that country and the disease become thereafter endemic in cattle and wildlife (Jori et al., 2016). In addition, due to multiple factors, including the increase of buffalo and elephant densities in some areas (Jori and Etter, 2016), the porosity of fences (Jori et al., 2009, 2011) and the low capacity of available vaccines (Thomson, 2008), all countries in the region have been experiencing an important increase in the number of FMD outbreaks to levels rarely experienced before (Thomson et al., 2013a; Jori et al., 2016).

#### Assessment of Utilizing Partial VP1 Sequences

To assess if using partial VP1-coding region sequences, compared to using whole VP1, would affect the estimation of the tree topology or ancestral character reconstruction, we repeated the analyses using a subset of 42 viruses for which full VP1 sequences were available. Clock model and tree prior selected in the previous step were used. Location and species characters were specified as described above.

## Results

Most of the sequences used (82%, *n* = 114) were collected in protected areas currently encompassing TFCAs, of which 80 and 34 corresponded to the GL TFCA and KAZA TFCA, respectively. Zimbabwe also was represented by a higher number of sequences (*n* = 14), compared to fewer samples from Botswana (*n* = 4), Namibia (*n* = 3) and Zambia (*n* = 4). Regarding species, the number of cattle and buffalo samples was similar, with 62 and 77 sequences, respectively (Supplementary Table S1).

### Phylogenetic Tree Prior Model Selection

The best partition and substitution models selected where codon position 1 + 2 substitution model TrN + I + G, and substitution model GTR + I + G for codon position 3. The model selected by AICM, and therefore used for the final analysis, was the exponential growth model (AICM = 14818). However, the AICM for the Bayesian Skyline was only slightly higher (AICM = 14888). The mean of the coefficient of variation in the final model was 0.93 (95%HPD 0.83–1.02), significantly different from 0, so the strict molecular clock was ruled out. The substitution rate per site per year estimated for the analyzed partial VP1 segment was 1.13 × 10^-2^ (95% HPD 8.61 × 10^-3^, 1.41 × 10^-2^). Specific substitution rates topotypes I, II, and III were 1.22 × 10^-2^ (95%HPD 9.01 × 10^-3^–1.57 × 10^-2^), 1.31 × 10^-2^ (95%HPD 9.42 × 10^-3^–1.71 × 10^-2^) and 1.28 × 10^-2^ (95%HPD 8.22 × 10^-3^–1.74 × 10^-2^) respectively.

### Discrete Traits for Host Species and Location

**FIGURE 1:**
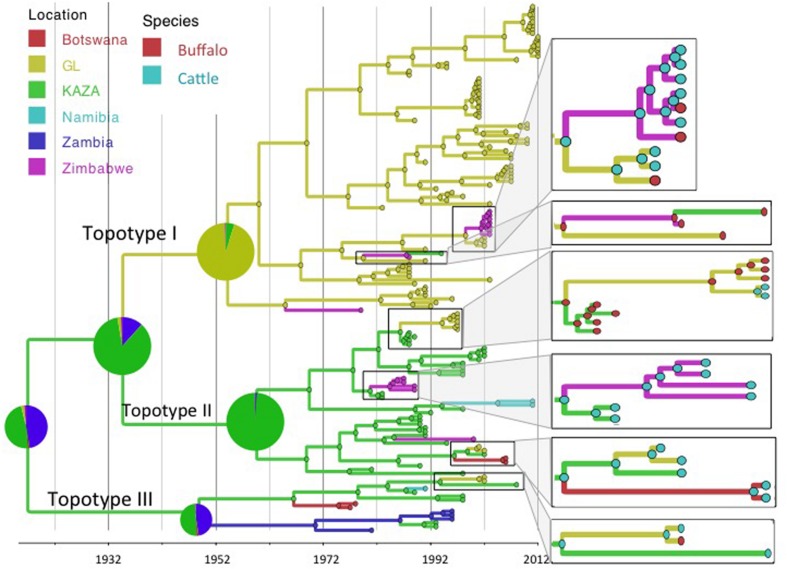
**Maximum clade credibility time-calibrated tree of FMDV SAT2 sequences analyzed.** Tree branches and node colors correspond to the location with the higher probability. Location probabilities of ancestors at the origins are depicted by a pie chart. Colors of nodes in the enlarged sections of the tree correspond to the species with the highest probability. Phylogeny was estimated using partial VP1 (384 nt) sequences.

**FIGURE 2:**
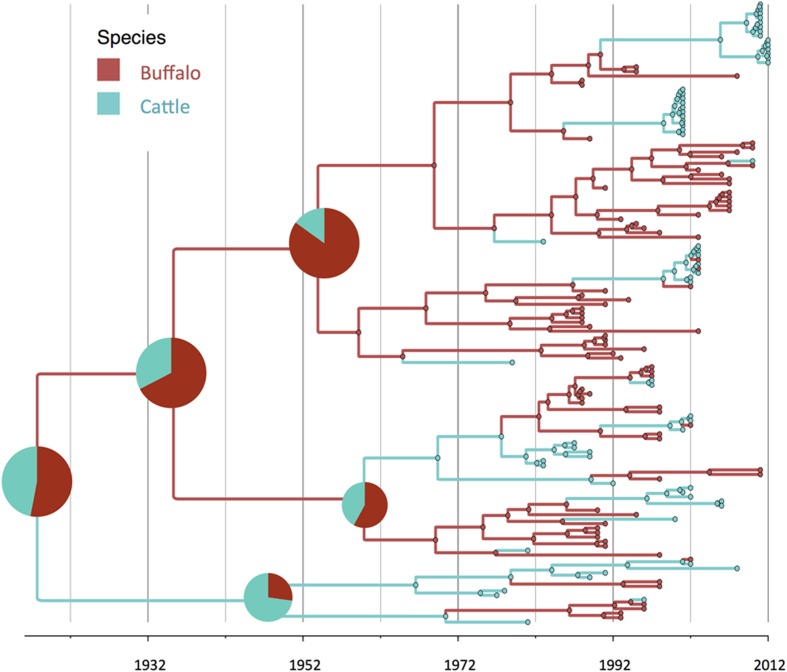
**Maximum clade credibility time-calibrated tree of FMDV SAT2 sequences analyzed.** Tree branches and node colors correspond to the species with the higher probability. Species probabilities of ancestors at the origins are depicted by a pie chart. Phylogeny was estimated using partial VP1 (384 nt) sequences.

Reconstruction of the FMD SAT2 phylogeny using the exponential growth tree prior and location and species as discrete traits is shown in **Figures 1** and **2**. FMDV serotype SAT2, topotypes I, II, and III as described by Knowles and Samuel (2003) are shown in **Figure 1**. Topotype I was mostly restricted to the GL TFCA area with three incursions into Zimbabwe in 1979 (95% HPD: 1970–1986), 1965 (95% HPD: 1952–1974), and 1999 (95%HPD: 1994–2001) and one further introduction into the current KAZA area in 1987 (95% HPD: 1985–1988), as evidenced by a virus isolated later in 1994. Topotypes II and III were geographically confined to the KAZA region and surrounding countries with some incursions of topotype II occurring into GL TFCA region in 1986 (95% HPD: 1985–1988) and again in 1999 (95% HPD: 1996–2001), into Zimbabwe in 1981 (95%HPD: 1977–1983) and in 1985 (95%HPD: 1976–1990), into Botswana in 1996 (95%HPD: 1991–2000) and into Namibia in 1994 (95%HPD: 1990–1997). Incursion of topotype III from KAZA into Zambia was evident in 1949 (95% HPD: 1920–1967), into Namibia in 1987 (1984–1989), into Botswana in 1967 (95% HPD: 1952–1974), and into GL 1994 (95% HPD: 1987–1999).

**FIGURE 3:**
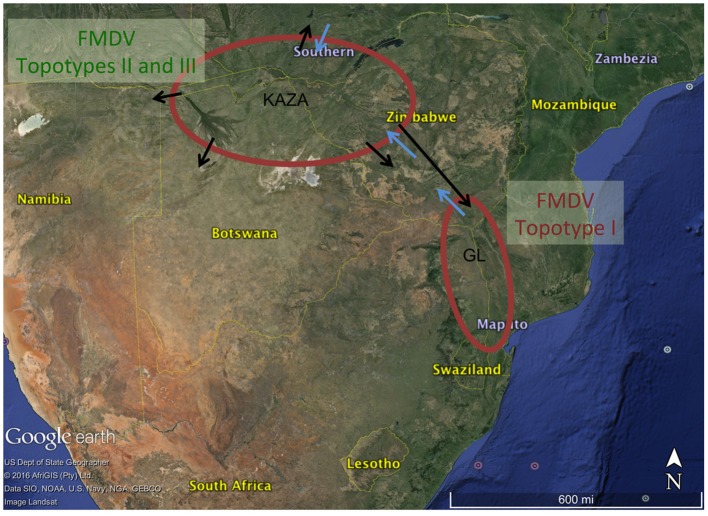
**Significant transmission between countries and TFCAs: Kavango–Zambezi (KAZA) and Great Limpopo (GL) TFCAs areas are indicated with a red circle.** Arrows represent significant transmission (BF > 3) as detected by BSSVS.

The BSSVS analysis used to determine significant transmission between locations showed strong evidence of transmission from both TFCAs to the respective countries in which they are circumscribed and from where outbreak samples were obtained (i.e., from KAZA TFCA into Zambia, Namibia, Botswana, and Zimbabwe, and from GL TFCA into Zimbabwe; **Figure 3**). There was no statistical support (BF < 3.0) of virus migration from GL TFCA to KAZA TFCA. In contrast, transmission from KAZA to GL TFCA was significant (**Table 1**). Topotype II phylogeny showed two transmission events from KAZA to GL TFCA: one of them in 1986 (95% HDP: 1985–1988) initially from buffalo, and detected in 1997 in infected buffalo and cattle in the GL TFCA (**Figure 1**). Another topotype II isolated in cattle was introduced into GL TFCA in 1999 (95%HPD: 1996–2001) shortly before being detected in 2001. This virus also caused outbreaks in cattle in Botswana in 2006.

**Table 1:** Bayes factor of transmission rates found using asymmetric Bayesian stochastic search variable selection (BSSVS) between locations and species.

	From	To	BF
Location	KAZA	GL	>1000
	GL	Zimbabwe	>1000
	KAZA	Namibia	>1000
	KAZA	Botswana	234.992
	Zimbabwe	KAZA	83.340
	KAZA	Zimbabwe	62.234
	Zambia	KAZA	17.668
	KAZA	Zambia	3.475
	Namibia	Zambia	1.394
	Namibia	Botswana	1.355
	Namibia	Zimbabwe	1.263
	Namibia	GLTP	1.214
	Namibia	KAZA	1.208
	Botswana	Zambia	1.191
	Botswana	KAZA	1.187
	Zambia	GLTP	1.116
	Botswana	Zimbabwe	0.977
	Botswana	Namibia	0.966
Species	Buffalo	Cattle	>1000
	Cattle	Buffalo	>1000

*Bayes factor > 3 was considered significant non-zero transmission.*

Geographically, results showed a high probability of topotypes I and II ancestors being originated in the current region of the KAZA TFCA area (*P* = 0.86) in 1935 (95% HPD: 1904–1957). The origins of topotype III showed a higher uncertainty, the ancestor’s most likely location being KAZA (*P* = 0.49), but other areas within Namibia had a similar probability (*P* = 0.47) of originating this topotype.

There was strong evidence supported by BSSVS analysis of transmission from buffalo to cattle, and from cattle to buffalo (**Table 1**). Origins of the topotypes within a specific host did show high uncertainty of the probability of buffalo or cattle being the host of viruses in early years of the tree reconstruction (**Figure 2**). Topotype II showed a higher uncertainty of the species origins approximately until late 1980’s. Topotype III on the other hand showed a high probability of being a virus initially circulating in cattle and later transmitted to buffalo.

Phylogenetic reconstruction using a subset of sequences with full VP1 available, revealed that results of the analysis using partial VP1 were similar to those obtained using partial VP1 (Supplementary Figure S1). The substitution rate per site per year estimated for the complete VP1 sequence was lower: 6.87 × 10^-3^ (2.70 × 10^-2^–1.14 × 10^-3^), compared to the estimated rate for the partial sequence: 1.13 × 10^-2^ (95% HPD 8.61 × 10^-3^, 1.41 × 10^-2^).

**FIGURE 4:**
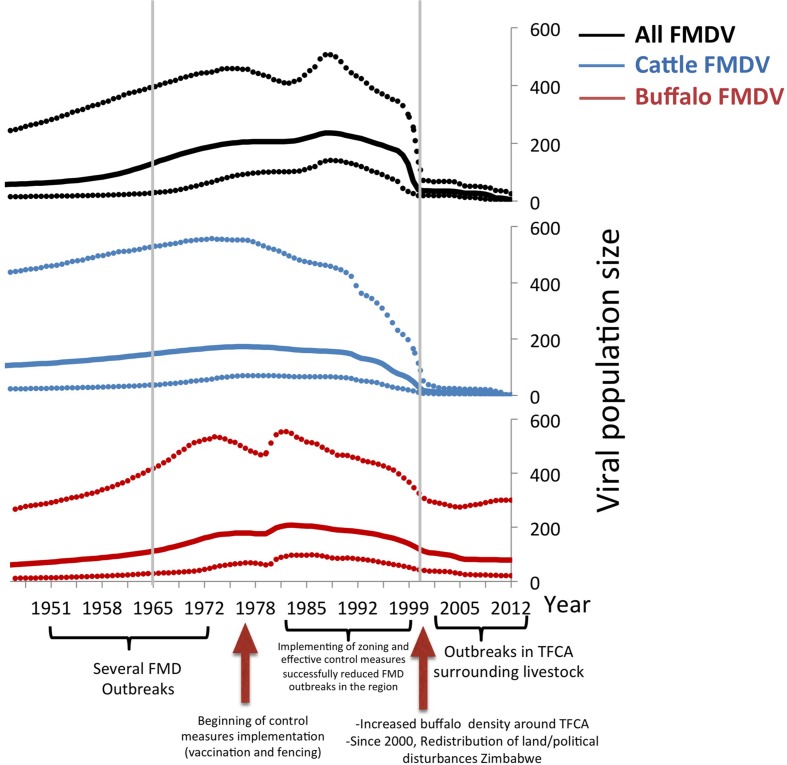
**Bayesian skyline plot of FMDV SAT2.** All sequences, buffalo sequences, and cattle sequences are depicted in blue, red, and green, respectively. The *x*-axis represents time, and the *y*-axis represents the estimated effective population size, which approximates viral diversity. The dotted lines represent the upper and lower 95% credibility intervals of the viral population size estimated by the Bayesian skyline demographic reconstruction. The three relevant periods of FMDV occurrence in Southern Africa (1951 to 1965, 1966 to1999, and 2000-current days), are indicated by gray lines.

Results of the viral population size of all FMDV, and viruses in cattle and buffalo are shown in **Figure 4**. There was an estimated increase of the viral population size until the mid 1970s, reaching a first peak that was mostly given by the peak of the viral diversity in cattle. During the following decade, the virus diversity slowly decreased in cattle but increased in buffalo. There was a steep fall of the virus diversity in both species previous to the year 2000, and a maintained low viral diversity in cattle until 2012, whereas virus diversity in buffalo was higher than that in cattle. Events related to FMD control and occurrence, which may have affected the effective viral population size, are indicated at the bottom of the Bayesian skyline plots (**Figure 4**). Although outbreaks in livestock in Southern Africa increased in early 2000s, and several outbreaks have been reported in Botswana and South Africa, the viral diversity dropped in the cattle population before this period and has remained low until the present. The genetic diversity of FMDV also decreased in the buffalo population, however, as expected, it has remained higher than the diversity observed in cattle.

## Discussion

Animal diseases in general and FMD in particular are known to circulate at the interface of any given protected area hosting buffalo populations in Sub-Saharan Africa. Viral transmission between TFCAs poses a complex scenario, because, animals co-existing with a certain FMDV strain develop immunity against that specific virus, but may be more susceptible to a different topotype, because of their likely antigenic differences (Bastos et al., 2001; Paton et al., 2005). Circulation of SAT 2 FMDV has been traditionally geographically circumscribed; while SAT 2 FMDV topotype I is found in the GL area, topotypes II and III occur in the KAZA area (**Figure 1**; Bastos et al., 2003). However, results from our study strongly support the scenario of sporadic FMDV SAT 2 migration from KAZA into GL, and from both TFCAs into nearby territories outside the TFCAs borders. Transmission of different FMDV topotypes between endemic areas, compromise the effective vaccine immunization if vaccine formulation does not include the appropriate antigens.

Our results suggest that FMDV-infected animals have likely been moved from the KAZA TFCA across Zimbabwe until reaching GL TFCA areas at least in three occasions (**Figure 3**). One of these events, has been previously described; viruses of topotype II, mainly found in buffalo from KAZA TFCA area were also found in buffalo in 1997 in a reserve close to the Zimbabwe area of Gonarezhou National Park, which currently encompasses the Zimbabwean side of the GL TFCA, and was further transmitted to livestock in surrounding areas (Hargreaves et al., 2004). Our results also suggest that introduction of topotype II into GL TFCA occurred again in 1998 (**Figure 1**), and was detected in cattle in 2001. This virus was subsequently found in cattle from Botswana within the KAZA interface, and later in 2006 it was found in cattle in areas of Botswana not related to KAZA. Additionally, FMDV topotype III identified for the first time in KAZA, was also found in cattle and buffalo of GL in Zimbabwe in 2002, having a likely epidemiological link. All these incursions of FMDV topotypes into their non-endemic areas have only caused local outbreaks, without reported data of becoming established or spreading beyond their original endemic areas.

The current endemic situation of FMD in Zimbabwe, which is home of the eastern area of KAZA TFCA and the northern area of GL TFCA, might have contributed to an adaptation of some FMDV strains to cattle. During the 2000–2002 period, Zimbabwe went under political instability and redistribution of land, after a period in which FMDV was relatively controlled. Such situation and the establishment of private game reserves with high number of buffalos, some of them translocated from FMD-infected areas, resulted in FMD becoming endemic, affecting all neighboring countries, especially those sharing TFCAs (Vosloo et al., 2006; Smitz et al., 2014).

Our results suggest that FMDV SAT2 topotype I, was transmitted in one occasion from GL TFCA into Zimbabwe and then into KAZA (**Figure 1**). Those viruses’ and their ancestors’ host were most likely buffalos. Another topotype I virus from GL TFCA apparently adapted to cattle, was further transmitted into buffalos from private Zimbabwean game reserves in Chinhoyi and Harare as well as buffalos and cattle in GL TFCA in 2002–2003. It is also important to note that FMDV topotypes I endemic to GL TFCA, and topotype II and III endemic to KAZA TFCA have transgressed between the TFCAs where they normally circulate, with no further reported consequences of becoming established and endemic in the newly introduced area, However, the potential for these viruses to spread further within naïve populations previously unexposed remains.

Regarding species ancestors, the probability of hosting the most recent common ancestor of all SAT2 viruses included in the study is not strongly supportive either for cattle or buffalo (probabilities close to 50%; **Figure 2**). Interestingly, results here strongly suggest that topotype III could have been a virus early adapted to cattle, and further spreading into buffalo in several occasions, although these results may have been biased by the high number of topotype III cattle samples, especially in early years. In a recent study, it has been suggested that it was buffalo the one that likely hosted the original virus, although using a different set of SAT2 samples (Hall et al., 2013).

Being able to relate the genetic diversity of the virus with the general epidemiological situation of FMD occurrence may help to predict FMD epidemics using genetic data. Results of the changes in the effective viral population size based on the sequences available can be related to the events of FMDV occurrence in South Africa. The pattern of viral population change analyzed in all SAT2 sequences available for our study is similar to that observed in buffalo samples alone (**Figure 4**). In general there is an increase of the viral population size in cattle until mid 1970s and in buffalo, which peaked in the mid 1980s and did not decreased drastically until mid to late 1990s. Control measures such as vaccination, and the erection of cordon fences that were implemented in the decades of the 1960s and 1970s, may be the reason why the virus population decreased during this period (**Figure 4**), however, the increased number of cattle samples (with low viral diversity) during the last decade can also influence this result. Furthermore although the viral population decreased in cattle, it continued to increase in buffalo. Starting in early 2000’s the, viral population showed a similar stationary trend rather than a decrease, in both cattle and buffalo samples.

After several years of disease freedom in meat exporting countries (Botswana, Namibia, and South Africa), FMD outbreaks have been consistently reported on a yearly basis since the early 2000s. This high incidence was likely due to an increased density of buffalo and cattle population, especially in areas surrounding conservation areas and changes in FMD control measures in Zimbabwe. Because FMDV is endemic to the buffalo population, where almost 100% seropositivity of healthy buffaloes sampled has been found (Thomson et al., 1992; Vosloo et al., 2002; Jori et al., 2016), most of these outbreaks at the interface of TFCAs have been attributed to buffalo from wildlife areas interacting with insufficiently immunized livestock (Sutmoller et al., 2000; Vosloo et al., 2002). Consistent with previous knowledge on the epidemiology of FMD in the region, our study supports transmission from buffalo into cattle; however, and most interesting, our results also support transmission from cattle into buffalo populations. This finding highlights the importance of livestock in the FMDV spread among cattle, between TFCA. Transmission and maintenance of disease among livestock may be a relevant event to consider in FMD epidemiology. Movements of animals or animal products can be responsible of the transportation and transmission of FMDV viruses within one country or even between different countries. This is even more likely in the case of clinically mild forms of the virus, which can travel long distances without being detected. Vaccination, quarantine and/or detection of infected livestock living or moving close to TFCA and sanitary control of buffaloes before being translocated between private game reserves into different areas in the country are crucial for preventing potential incursions of new FMD topotypes outside their original range.

Results in this study may have been influenced by biases associated with the non-random selection of samples, and potential under sampling of certain location and species. This limitation is common among phylogenetic studies, due to a non-random availability of sequences either from public databases or from diagnostic laboratories (Frost et al., 2015). In this case, sampling activities were conducted under surveillance and research activities, which normally depends on available economic resources that are not perennial. Because the African buffalo is known to be the natural reservoir and to have high disease prevalence, future studies should aim at obtaining regular samples from animals at different locations in order to contribute to a more complete reconstruction of the viral phylogeny at a regional level.

Assessment of analysis made with partial VP1 sequences have shown almost identical results of those obtained by using whole VP1. A more structured sampling and information of viral sequences may provide more accurate information to understand virus phylodynamics. However, in practice feasibility of such studies is limited by budget and logistics constrains.

## Conclusion

Results from our study suggest that, in addition to buffalo, cattle may also play an important role in long distance spread of FMDV, facilitating migration and mixing of topotypes, which can reduce vaccine matching and compromise adequate immunization and success of vaccination campaigns. Early detection, immunization, and prevention of effective inter-species transmission should be stringent in order to control FMDV. Sporadic transmission between TFCAs stresses the need for implementation of control measures not only at a local level but also with a regional approach that should include the collaboration of all countries involved in the management of TFCAs.

## Author Contributions

Conceived and designed the study: AP, FJ, FM, BB. Viral sequencing: LH, RD, FM. Data compilation: LH, RD. Sequence analysis: BB. Wrote the paper: BB, FJ, FM, AP.

## Conflict of Interest Statement

The authors declare that the research was conducted in the absence of any commercial or financial relationships that could be construed as a potential conflict of interest.
