# Supplementary material for: Transmission of Foot-and-Mouth Disease SAT2 Viruses at the Wildlife–Livestock Interface of Two Major Transfrontier Conservation Areas in Southern Africa
Source: Front Microbiol. 2016 Apr 22;7:528. doi: 10.3389/fmicb.2016.00528 (PMC4840674; doi:10.3389/fmicb.2016.00528)
Supplement: Supplementary file 1 [file Table_1.DOCX]

**Transmission of foot-and-mouth disease SAT2 viruses at the wildlife-livestock interface of two major transfrontier conservation areas**

Barbara P. Brito*^1^, Ferran Jori^2,3,4^, Rahana Dwarka^5^, Francois Maree^3,6^, Livio Heath^5^ and Andres M. Perez^6^

*** Correspondance:** Dr. Barbara Brito, Universidad de Chile, Departamento de Medicina Preventiva Animal, Facultad de Ciencias Veterinarias y Pecuarias, Av. Santa Rosa 11735, La Pintana, Santiago, Chile

barbara.brito.r@gmail.com

Supplementary Table 1. Sequences with corresponding location and host data used for Phylogenetic analysis.

| **Name** | **Year** | **Host** | **Country** | **Location** | **Area** | **GenBank Accesion Number** |
| --- | --- | --- | --- | --- | --- | --- |
| ZIM/07/83 | 1983 | Cattle | Zimbabwe | Nyamandlovu | KAZA | AF023523 |
| ZIM/1/97 | 1997 | Cattle | Zimbabwe | Masvingo, Chiredzi | GL | AF136981 |
| ZIM/4/97 | 1997 | Cattle | Zimbabwe | Masvingo, Chiredzi | GL | AF136982 |
| ZIM/42/97 | 1997 | Buffalo | Zimbabwe | Mukazi Ranch, Chiredzi | GL | AF136983 |
| ZIM/44/97 | 1997 | Buffalo | Zimbabwe | Mukazi Ranch, Chiredzi | GL | AF136984 |
| ZIM/48/97 | 1997 | Buffalo | Zimbabwe | Mukazi Ranch, Chiredzi | GL | AF136985 |
| KNP/8/88 | 1988 | Buffalo | South Africa | Rietpan | GL | AF137001 |
| KNP/9/88 | 1988 | Buffalo | South Africa | Shilolweni | GL | AF137002 |
| KNP/10/88 | 1988 | Buffalo | South Africa | Shilolweni | GL | AF137003 |
| KNP/14/88 | 1988 | Buffalo | South Africa | Ripape | GL | AF137004 |
| KNP/25/89 | 1989 | Buffalo | South Africa | Matjipiri | GL | AF137006 |
| KNP/40/89 | 1989 | Buffalo | South Africa | Ripape | GL | AF137007 |
| KNP/143/91 | 1991 | Buffalo | South Africa | Reënvoëldam | GL | AF137009 |
| KNP/147/9 | 1991 | Buffalo | South Africa | Reenvoel Dam | GL | AF137010 |
| KNP/160/91 | 1991 | Buffalo | South Africa | Ndziyo Spruit | GL | AF137011 |
| KNP/9/93 | 1993 | Buffalo | South Africa | Boyelaspruit | GL | AF137014 |
| KNP/16/93 | 1993 | Buffalo | South Africa | Capricorn | GL | AF137015 |
| KNP/6/96 | 1996 | Buffalo | South Africa | Mhalangangwane Dam | GL | AF137018 |
| KNP/24/96 | 1996 | Buffalo | South Africa | Mulalanespruit | GL | AF137019 |
| PAL/5/83 | 1983 | Cattle | South Africa | - | GL | AF367102 |
| KNP/07/88 | 1988 | Buffalo | South Africa | Rietpan | GL | AF367103 |
| KNP/7/88 | 1988 | Buffalo | South Africa | Rietpan | GL | AF367103 |
| ZIM/1/88 | 1988 | Buffalo | Zimbabwe | Hwange National Park | KAZA | AF367108 |
| KNP/32/92 | 1992 | Buffalo | South Africa | Boyela Vlakteplaas | GL | AF367113 |
| ZIM/Gn10/91 | 1991 | Buffalo | Zimbabwe | Gonarezhou | GL | AF367113 |
| ZAM/9/93 | 1993 | Buffalo | Zambia | Nanzhila, Kafue National Park | KAZA | AF367116 |
| ZAM/10/93 | 1993 | Buffalo | Zambia | Nanzhila, Kafue National Park | KAZA | AF367117 |
| KNP/18/95 | 1995 | Buffalo | South Africa | Mondzweni | GL | AF367118 |
| KNP/31/95 | 1995 | Buffalo | South Africa | Mondzweni | GL | AF367119 |
| ZAM/7/96 | 1996 | Buffalo | Zambia | Mulanga | Zambia | AF367120 |
| ZAM/10/96 | 1996 | Cattle | Zambia | Buff 10. Mulanga | Zambia | AF367121 |
| BOT/1/98 | 1998 | Buffalo | Botswana | Nxaraga | KAZA | AF367122 |
| BOT/18/98 | 1998 | Buffalo | Botswana | Nxaraga | KAZA | AF367123 |
| BOT/29/98 | 1998 | Buffalo | Botswana | Vumbura | KAZA | AF367124 |
| BOT/31/98 | 1998 | Buffalo | Botswana | Vumbura | KAZA | AF367125 |
| NAM/286/98 | 1998 | Buffalo | Namibia | Mouma, E Caprivi Game Reserve | KAZA | AF367127 |
| NAM/292/98 | 1998 | Buffalo | Namibia | Mouma, E Caprivi Game Reserve | KAZA | AF367128 |
| NAM/304/98 | 1998 | Buffalo | Namibia | Mahango, W Caprivi Game Park | KAZA | AF367129 |
| ZIM/267/98 | 1998 | Buffalo | Zimbabwe | Chizarira | KAZA | AF367130 |
| ZIM/9/88 | 1988 | Buffalo | Zimbabwe | - | Zimbabwe | AY254711 |
| RHO/2/79 | 1979 | Cattle | Zimbabwe | Chiredzi | Zimbabwe | AY254713 |
| ZIM/2/88 | 1988 | Buffalo | Zimbabwe | Hwange National Park | KAZA | AY254715 |
| ZIM/4/88 | 1988 | Buffalo | Zimbabwe | Hwange National Park | KAZA | AY254716 |
| ZIM/7/89 | 1989 | Buffalo | Zimbabwe | Hwange National Park | KAZA | AY254719 |
| NAM/1/91 | 1991 | Cattle | Namibia | - | Namibia | AY254720 |
| ZIM/Gn3/91 | 1991 | Buffalo | Zimbabwe | Gonarezhou | GL | AY254721 |
| ZIM/16/91 | 1991 | Buffalo | Zimbabwe | Matusadona National Park | KAZA | AY254722 |
| ZIM/34/91 | 1991 | Buffalo | Zimbabwe | Urungwe Safari Area | KAZA | AY254723 |
| NAM/01/92 | 1992 | Cattle | Namibia | 80KM E Of Rundu, Kavango | KAZA | AY254724 |
| ZIM/08/94 | 1994 | Buffalo | Zimbabwe | Matetsi | KAZA | AY254725 |
| KNP/46/95 | 1995 | Buffalo | South Africa | Kruger National Park | GL | AY254726 |
| ZIM/7/95 | 1995 | BUFFALO | Zimbabwe | Sengwa | KAZA | AY254727 |
| ZAM/8/96 | 1996 | Buffalo | Zambia | Mulanga | Zambia | AY254728 |
| SAR/1/01 | 2001 | Cattle | South Africa | Orinoco | GL | AY442903 |
| SAR/2/01 | 2001 | Cattle | South Africa | Orinoco | GL | AY442904 |
| SAR/3/01 | 2001 | Cattle | South Africa | Orinoco | GL | AY442905 |
| SAR/4/01 | 2001 | Cattle | South Africa | Orinoco | GL | AY442906 |
| SAR/5/01 | 2001 | Cattle | South Africa | Orinoco | GL | AY442907 |
| SAR/6/01 | 2001 | Cattle | South Africa | Newington | GL | AY442908 |
| SAR/7/01 | 2001 | Cattle | South Africa | Dwarsloop | GL | AY442909 |
| SAR/8/01 | 2001 | Cattle | South Africa | Dwarsloop | GL | AY442910 |
| SAR/9/01 | 2001 | Cattle | South Africa | Dwarsloop | GL | AY442911 |
| SAR/10/01 | 2001 | Cattle | South Africa | Craigieburn | GL | AY442912 |
| SAR/11/01 | 2001 | Cattle | South Africa | Craigieburn | GL | AY442913 |
| ZIM/17/91 | 1991 | Buffalo | Zimbabwe | Urungwe Safari Area | KAZA | DQ009727 |
| ZIM/14/90 | 1990 | Buffalo | Zimbabwe | Doma Safari Area | KAZA | DQ009728 |
| ZIM/34/90 | 1990 | Buffalo | Zimbabwe | Chirisa Safari Area | KAZA | GU194490 |
| ZIM/5/83 | 1983 | Cattle | Zimbabwe | - | KAZA | JQ639289/DQ009726 |
| ZIM/13/01 | 2001 | Cattle | Zimbabwe | Lupane Area (Jotholo) | KAZA | JQ639292 |
| KNP/3/10 | 2010 | Buffalo | South Africa | Pafuri Area | GL | JQ950548 |
| KNP/2/10 | 2010 | Buffalo | South Africa | Pafuri Area | GL | JQ950550 |
| KNP/1/10 | 2010 | Buffalo | South Africa | Pafuri Area | GL | JX088744 |
| BOT/3/77 | 1977 | Cattle | Botswana | - | Botswana | KF112928 |
| BOT08/78 | 1978 | Cattle | Botswana | - | Botswana | KF112929 |
| SWA/4/89 | 1989 | Cattle | Namibia | Sigwe Village, East Caprivi | KAZA | KF112969 |
| ZAM/3/81 | 1981 | Cattle | Zambia | - | Zambia | KF112971 |
| ZIM/5/81 | 1981 | Cattle | Zimbabwe | - | KAZA | KF112972 |
| ZIM/1/87 | 1987 | Cattle | Zimbabwe | Insiza | Zimbabwe | KF112973 |
| ZIM/5/87 | 1987 | Cattle | Zimbabwe | Masvingo | Zimbabwe | KF112974 |
| ZIM/8/89 | 1989 | Cattle | Zimbabwe | Mutorashanga | Zimbabwe | KF112975 |
| ZIM/9/89 | 1989 | Cattle | Zimbabwe | Gweru | Zimbabwe | KF112976 |
| MOZ/1/10 | 2010 | Cattle | Mozambique | Bilene, Chokwe | GL | Q950549 |
| BOT/13/02 | 2002 | Cattle | Botswana | Matseloje Area | KAZA | KU994773 |
| BOT/4/06 | 2006 | Cattle | Botswana | Thabana | Botswana | KU994774 |
| BOT/5/06 | 2006 | Cattle | Botswana | Nala | Botswana | KU994775 |
| KNP/04/03 | 2003 | Buffalo | South Africa | Tshamavadzi, Punda Maria | GL | KU994776 |
| KNP/05/03 | 2003 | Buffalo | South Africa | Lower Sabie, Punda Maria | GL | KU994777 |
| KNP/06/03 | 2003 | Buffalo | South Africa | Wik En Weeg, Shangoni | GL | KU994778 |
| KNP/1/06 | 2006 | Buffalo | South Africa | Masakosa | GL | KU994779 |
| KNP/11/07 | 2007 | Buffalo | South Africa | Shingwedzi Area, Bububu | GL | KU994780 |
| KNP/12/08 | 2008 | Buffalo | South Africa | Lower Sabie Area | GL | KU994781 |
| KNP/141/91 | 1991 | Buffalo | South Africa | Kruger National Park | GL | KU994782 |
| KNP/15/07 | 2007 | Buffalo | South Africa | Shingwedzi Area, Shingumeni | GL | KU994783 |
| KNP/16/07 | 2007 | Buffalo | South Africa | Shingwedzi Area, Shingumeni | GL | KU994784 |
| KNP/1678/98 | 1998 | Buffalo | South Africa | Bume, Kruger National Park | GL | KU994785 |
| KNP/18/07 | 2007 | Buffalo | South Africa | Shingwedzi Area, Shingumeni | GL | KU994786 |
| KNP/20/07 | 2007 | Buffalo | South Africa | Shingwedzi Area, Langtoondam | GL | KU994787 |
| KNP/25/07 | 2007 | Buffalo | South Africa | Shingwedzi Area, Boyela | GL | KU994788 |
| KNP/4/07 | 2007 | Buffalo | South Africa | Shingwedzi Area, Dzombo | GL | KU994789 |
| KNP/5/06 | 2006 | Buffalo | South Africa | Nwambi Block | GL | KU994790 |
| KNP/9/03 | 2003 | Buffalo | South Africa | Ribye Waterhole | GL | KU994791 |
| KNP/9/08 | 2008 | Buffalo | South Africa | Lower Sabie Area | GL | KU994792 |
| NAM/1/08 | 2008 | Cattle | Namibia | Katimu Malilo | KAZA | KU994793 |
| NAM/3/10 | 2011 | Buffalo | Namibia | Khaiseb/Jaco | Namibia | KU994794 |
| NAM/5/10 | 2011 | Buffalo | Namibia | Khaiseb/Jaco | Namibia | KU994795 |
| SAR/11/1919/11 | 2011 | Cattle | South Africa | Kruger National Park | GL | KU994796 |
| SAR/12/0050/12 | 2012 | Cattle | South Africa | Kruger National Park | GL | KU994797 |
| SAR/12/0157/12 | 2012 | Cattle | South Africa | Kruger National Park | GL | KU994798 |
| SAR/12/0208/12 | 2012 | Cattle | South Africa | Kruger National Park | GL | KU994799 |
| SAR/12/0209/12 | 2012 | Cattle | South Africa | Kruger National Park | GL | KU994800 |
| SAR/12/0284/12 | 2012 | Cattle | South Africa | Kruger National Park | GL | KU994801 |
| SAR/12/0331/12 | 2012 | Cattle | South Africa | Kruger National Park | GL | KU994802 |
| SAR/1801/1/11 | 2011 | Cattle | South Africa | Kruger National Park | GL | KU994803 |
| SAR/1801/2/11 | 2011 | Cattle | South Africa | Kruger National Park | GL | KU994804 |
| SAR/1801/3/11 | 2011 | Cattle | South Africa | Kruger National Park | GL | KU994805 |
| SAR/1801/4/11 | 2011 | Cattle | South Africa | Kruger National Park | GL | KU994806 |
| SAR/1840/1/11 | 2011 | Cattle | South Africa | Kruger National Park | GL | KU994807 |
| SAR/1840/2/11 | 2011 | Cattle | South Africa | Kruger National Park | GL | KU994808 |
| SAR/1840/3/11 | 2011 | Cattle | South Africa | Kruger National Park | GL | KU994809 |
| ZIM/01/00 | 2000 | Cattle | Zimbabwe | Elephant Walk Tengwe Farm | Zimbabwe | KU994810 |
| ZIM/01/02 | 2002 | Cattle | Zimbabwe | Beitbrug Area | GL | KU994811 |
| ZIM/05/02 | 2002 | Cattle | Zimbabwe | Lupane Area (Jotholo) | KAZA | KU994812 |
| ZIM/06/02 | 2002 | Cattle | Zimbabwe | Lupane Area (Jotholo) | KAZA | KU994813 |
| ZIM/08/02 | 2002 | Buffalo | Zimbabwe | Lupane Area (Jotholo) | KAZA | KU994814 |
| ZIM/09/02 | 2002 | Buffalo | Zimbabwe | Bikita | GL | KU994815 |
| ZIM/1/03 | 2003 | Cattle | Zimbabwe | Harare South | Zimbabwe | KU994816 |
| ZIM/10/02 | 2002 | Cattle | Zimbabwe | Gumira Dt, Ndowoyo Area | GL | KU994817 |
| ZIM/10/03 | 2003 | Buffalo | Zimbabwe | Manicaland, Chinhoyi | Zimbabwe | KU994818 |
| ZIM/11/01 | 2001 | Cattle | Zimbabwe | Beitbrug Area | GL | KU994819 |
| ZIM/11/02 | 2002 | Buffalo | Zimbabwe | Bikita | GL | KU994820 |
| ZIM/12/02 | 2002 | Cattle | Zimbabwe | Top Camp Dt, Bikita | GL | KU994821 |
| ZIM/12/90 | 1990 | Buffalo | Zimbabwe | Doma Safari Area | KAZA | KU994822 |
| ZIM/13/02 | 2002 | Cattle | Zimbabwe | Top Camp Dt, Bikita | GL | KU994823 |
| ZIM/20/90 | 1990 | Buffalo | Zimbabwe | Chirisa Safari Area | KAZA | KU994824 |
| ZIM/4/03 | 2003 | Cattle | Zimbabwe | Harare | Zimbabwe | KU994825 |
| ZIM/5/03 | 2003 | Cattle | Zimbabwe | Harare | Zimbabwe | KU994826 |
| ZIM/6/03 | 2003 | Buffalo | Zimbabwe | Harare | Zimbabwe | KU994827 |
| ZIM/7/03 | 2003 | Cattle | Zimbabwe | Chinhoyi | Zimbabwe | KU994828 |
| ZIM/9/03 | 2003 | Cattle | Zimbabwe | Harare | Zimbabwe | KU994829 |
