# Supplementary material for: Transmission of Foot-and-Mouth Disease SAT2 Viruses at the Wildlife–Livestock Interface of Two Major Transfrontier Conservation Areas in Southern Africa
Source: Front Microbiol. 2016 Apr 22;7:528. doi: 10.3389/fmicb.2016.00528 (PMC4840674; doi:10.3389/fmicb.2016.00528)
Supplement: Supplementary file 2 [file Image_1.PDF]

Transmission of foot-and-mouth disease SAT2 viruses at the wildlife-livestock interface of two major transfrontier conservation areas

Barbara P. Brito*[1], Ferran Jori[2,3,4], Rahana Dwarka[5], Francois Maree[3,6], Livio Heath[5] and Andres M. Perez[6]

*** Correspondance:** Dr. Barbara Brito, Universidad de Chile, Departamento de Medicina Preventiva Animal, Facultad de Ciencias Veterinarias y Pecuarias, Av. Santa Rosa 11735, La Pintana, Santiago, Chile
barbara.brito.r@gmail.com

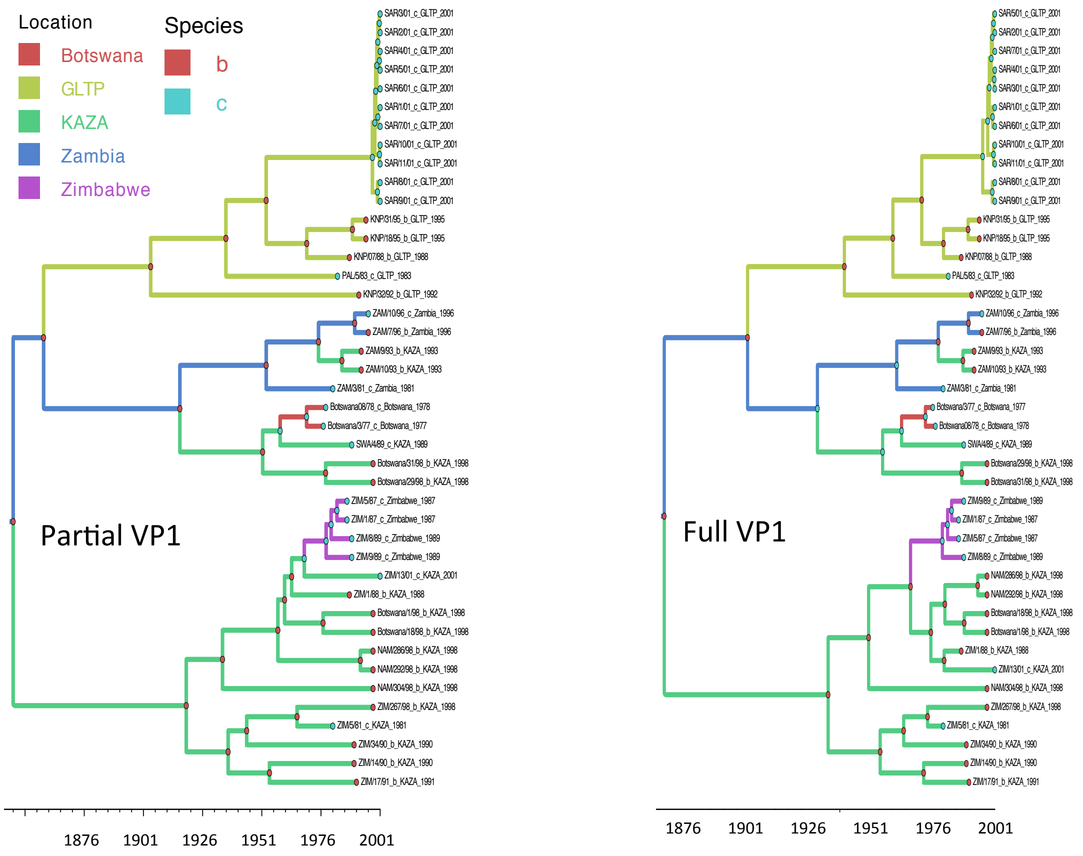

Image S1. The left panel depicts the MCC tree of FMDV SAT2 phylogeny reconstructed using a subset of virus with available full VP1 sequencing. MCC tree on the left panel shows the MCC tree reconstructed using only partial VP1 sequences of the same viruses.
